# Inhibition of flavohemeproteins enhances the emission and level of nitric oxide in barley root tips

**DOI:** 10.1007/s00709-025-02058-w

**Published:** 2025-04-01

**Authors:** Loriana Demecsová, Ľubica Liptáková, Katarína Valentovičová, Veronika Zelinová, Ladislav Tamás

**Affiliations:** Institute of Botany, Plant Science and Biodiversity Centre, Slovak Academy of Sciences, Dúbravská Cesta 9, 84523 Bratislava, Slovak Republic

**Keywords:** Flavohemeproteins, NO catabolism, NO emission, Plasma membrane electron transport chain

## Abstract

In this study, using a pharmaceutical approach, we analyzed the NO accumulation and emission from the root tips of barley seedlings and the possible mechanisms of NO catabolism. Application of flavohemeprotein inhibitors, such as azide, cyanide, diphenyleneiodonium and dicumarol, an inhibitor of the plasma membrane electron transport chain, increased the NO level in root tissue and stimulated the NO emission from root tip cells. It can be concluded that barley root tips generate and, at the same time, consume a considerable amount of NO, probably by the plasma membrane flavohemeproteins. This high NO-consuming activity of barley root tips efficiently degraded even the externally applied high concentrations of NO without marked root growth inhibition. These results suggest that the root tip cells NO consumption activity plays an important role in the regulation of NO level in barley root tips.

**Supplementary Information:**

The online version contains supplementary material available at 10.1007/s00709-025-02058-w.

## Introduction

Nitric oxide (NO) is a gaseous molecule with signaling properties both in prokaryotes and eukaryotes. Although this free radical has been described as a participant in many physiological processes, the main source of NO in higher plants still eludes detection (Neill et al. 2003; Domingos et al. 2015; Kolbert et al. 2019). A growing body of evidence indicates the involvement and cooperation of multiple pathways in plant NO generation, depending on the developmental stage, tissue and organ type and on the environmental conditions. In the past decades, numerous potential non-enzymatic and enzymatic NO synthetic pathways, localized into different cell compartments, have been described in plants (Astier et al. 2018; León and Costa-Broseta 2020).

It has long been observed that plant leaves under specific conditions (at acidic pH) are capable of liberating NO, probably via non-enzymatic chemical reduction of atmospheric nitrogen dioxide (Nishimura et al. 1986). It has been shown later that, during light, carotenoids are responsible for the conversion of toxic nitrogen dioxide to NO (Cooney et al. 1994). A non-enzymatic mechanism for the synthesis of NO from nitrite has also been suggested in the acidic apoplastic space of plant tissues in the presence of phenolic compounds or ascorbate (Stöhr and Ullrich 2002; Bethke et al. 2004).

In animals, the enzymatic generation of NO from arginine catalyzed by NO synthase (NOS) is the primary source of NO. Even though no typical NOS sequences have been identified in land plants, the applications of mammalian NOS inhibitors reduced the detected NO levels in several experiments (Corpas et al. 2009; Jeandroz et al. 2016). Therefore, the NOS-like activity in plants is still considered as a possible NO source. Constitutive arginine-dependent NOS activity was detected in different organs of pea seedlings, although the strongest NO generation was observed in the vascular tissue and epidermal cells (Corpas et al. 2006). On the subcellular level, NOS activity was localized into peroxisomes and similarly to animal NOS, it was strictly dependent on NADPH, calmodulin and required calcium (Corpas et al. 2004). On the other hand, arginine as a substrate involved in NO generation has been suggested through the biosynthesis and catabolism pathways of polyamines (Recalde et al. 2021). For a long time, nitrate reductase (NR) was the most analyzed enzyme responsible for NO synthesis due to the fact that it can produce NO in vitro, and because NR activity mutants have an altered NO synthesis (Desikan et al. 2002; Neill et al. 2003). In chloroplast, both the electron transport system-mediated nitrite-dependent and arginine-dependent NOS-like activity-catalyzed NO generation was indicated by in vitro exposure assay (Jasid et al. 2006).

Recently, it became well documented that besides the conversion of nitrate to nitrite, NR can mediate transfer of electrons from NAD(P)H to several oxidoreductases, including nitrite reductases (Chamizo-Ampudia et al. 2017). Therefore, NR can be involved in both regulation and synthesis of NO in plants. In addition, increasing evidence indicates that the bioactivation of nitrite in mammals, and generation of NO and other nitrogen species from nitrite by numerous metalloproteins, plays an important role in NO signaling (Amdahl et al. 2020). Several molybdenum enzymes were found to be able to reduce nitrite to NO, such as mitochondrial amidoxime reducing component, sulfite reductase, aldehyde reductase and xanthine oxidoreductase (Bender and Schwarz 2018). Apart from these molybdenum enzymes, hemeproteins also can generate NO from nitrite, mainly under anoxic and acidic conditions (Kim-Shapiro and Gladwin 2014). Several mitochondrial electron transport chain (mETC) components (cytochrome c oxidase, cytochrome c, complex III, alternative oxidase) are capable of reducing nitrite to NO, which under anoxic conditions allows, in a limited extent, NAD(P)H oxidation and ATP synthesis (Igamberdiev et al. 2010; Gupta and Igamberdiev 2011).

Moreover, the level of NO is dependent also on the rate of NO metabolism and scavenging, which is controlled by several different molecules and proteins. NO can react with reduced glutathione, producing S‐nitrosoglutathione, which acts as a NO reservoir and as an efficient donor for protein nitrosylation (Corpas et al. 2013; Frungillo et al. 2014). One of the most rapid reactions in biological systems occurs between NO and superoxide, leading to peroxynitrite formation, with a potential signaling function in plants (Arasimowicz-Jelonek and Floryszak-Wieczorek 2011). Hemeproteins are not involved only in NO production; they can also be NO scavengers. In microorganisms, NO is detoxified by NO dioxygenase (NOD), a flavohemoglobin possessing NAD(P)H and oxygen-dependent enzymatic activity converting NO to nitrate (Gardner et al. 1998; Bonamore and Boffi 2008). Moreover, a similar oxygen-dependent NOD activity was also described in mammalian cells catalyzed probably by flavin- and heme-containing enzymes (Gardner et al. 2001; Hallstrom et al. 2004; Schmidt and Mayer 2004). In plants, non-symbiotic hemoglobins (phytoglobins) play a key function in the enzymatic scavenging of NO (Perazzolli et al. 2006). Seed-specific hemoglobin in sugar beet exhibited high NOD activity and efficiently generated nitrate from NO during seed germination (Eriksson et al. 2019). Under hypoxia, the main function of plant cytosolic hemoglobins is to metabolize NO, originating from mitochondrial nitrite reduction, back to nitrate. This metabolic pathway is known in plant cells as the hemoglobin-NO cycle (Hebelstrup et al. 2007). In mammalian cells, NO was found to be a substrate for multiple members of the peroxidase superfamily under physiological conditions (Abu-Soud and Hazen 2000). Similarly to peroxidases, in the presence of lipid substrate, animal and human lipoxygenases catalytically consume NO (Coffey et al. 2001). Moreover, it has been demonstrated in vitro that NO is a good substrate for horse radish peroxidase compounds I and II (Glover et al. 1999).

Barley is the fourth most important cereal crop cultivated widely across the world, according to production quantity (Newton et al. 2011). In addition, due to its several essential features, barley is widely used as a research material in various studies (Saisho and Takeda 2011). It has been previously reported that alfalfa root cultures expressing barley hemoglobin, acting in concert with flavoproteins, effectively metabolize NO to nitrate, utilizing NADH as the electron donor (Igamberdiev et al. 2004). Moreover, barley seedlings effectively incorporate atmospheric NO as a nitrogen source (Zhang et al. 2019). Thus, it is possible that most of the NO, potentially released into the intercellular spaces, is also catabolized by cells to restrict the NO emission into the surroundings.

Therefore, the aim of this study was the NO emission analysis from the root tips of barley seedlings and the examination of possible mechanisms of NO catabolism. Using a pharmaceutical approach, we applied various inhibitors of flavohemeproteins and plasma membrane electron transport chain (PM-ETC), a transport system of electrons from electron donors to several plasma membrane flavohemeproteins. The obtained results suggest a considerable NO consumption activity of cells in the barley root tips, which probably, besides the NO production, is a key mechanism involved in the regulation of NO level in barley root tips.

## Materials and methods

### Plant material and growth conditions

Barley (*Hordeum vulgare* L*.* cv. Levitus obtained from Slovakia, Sládkovičovo-Nový Dvor, Plant Breeding Station, Hordeum Ltd) seeds were briefly rinsed (1–2 min) in distilled water, and afterwards germinated in the dark between distilled water-wetted filter paper for 20 h at the temperature of 22 ± 2 °C. Seeds with a visible protrusion of radicle were transferred to rectangle trays and arranged into rows between two sheets of moist filter paper. These trays were then placed in a nearly vertical position (so the root growth is guided downwards) and were kept moist from a reservoir of distilled water using a wick of filter paper. Three days after germination and incubation in the dark at 22 ± 2 °C, when primary root length reached approximately 4–5 cm, the seedlings were used for experiments.

In the case of indole-3-acetic acid (IAA) treatment, roots of intact seedlings were immersed for 30 min into distilled water (control) or into a 10 µM IAA solution (from 100 mM stock in DMSO; final concentration of DMSO in the incubation medium was 0.1%). Following the treatment, the seedlings were rinsed in distilled water for 5 min, followed by their incubation between two sheets of filter paper moistened with distilled water for 3 h and then were used for the experiments.

### Root growth analysis

To determine the effect of NO donor on root growth increment, the roots underwent 30 min treatments, during which the roots of seedlings were immersed into distilled water (controls) or into 0.1 mM GSNO (S-nitroso-L-glutathione). After these short-term treatments, the roots were rinsed in distilled water for 5 min and incubated for 6 h in a vertically oriented tray between two sheets of filter paper, as described above. The position of each seedling's longest root tip was marked on the filter paper at the beginning of incubation. Following the 6 h of incubation, the increment in root length was measured by an image analyzer after the root tips were excised at the marked place on the filter paper. The values are the means of five independent experiments (30 roots per experiment).

### Extracellular NO analysis and localization in the root tips

NO emission from the root tips was measured by cell-impermeable fluorescent probes DAF-FM (4-amino-5-methylamino- 2´,7´-difluorofluorescein) and DAR-4 M (diaminorhodamine-4 M). Four millimeters-long apical segments of each seedling's two longest roots (15 segments per reaction) were used for analysis. First, the root segments were washed for 5 min in 20 mM sodium phosphate buffer, pH 6.0, then these segments were incubated for 30 min at 30 °C with shaking in a 400 µL of reaction mixture containing 20 mM sodium phosphate buffer (pH 6.0), a fluorescent probe 5 µM DAF-FM or DAR-4 M (from 5 mM DMSO stock solution) and depending on the treatment chemicals with different effect listed below. NO scavenger, cPTIO (2-(4-carboxyphenyl)−4,4,5,5-tetramethylimidazoline-1-oxyl-3-oxide), was added into the reaction mixture to control the NO specificity of the reaction, at 0.1, 0.25, 0.5 and 1 mM concentrations. The NO donor, GSNO, was used at 0.1 mM concentration. The GSNO was freshly synthetized at 1 mM concentration by mixing equal volumes of GSH (2 mM) and NaNO_2_ (2 mM) prepared in 0.1 N HCl. After stirring the reaction mixture for 10 min, the pH was adjusted to 6.0 with NaOH (Broniowska et al. 2013). Inhibitors of various enzymes, including heme-containing proteins, potassium cyanide and sodium azide, were applied at concentrations from 0.001 to 1 mM. DPI (diphenyleneiodonium; from 10 mM stock in DMSO), an inhibitor of flavoproteins, was used at 0.1, 1, 10 and 100 µM concentrations. Dicumarol (from 10 mM stock in DMSO), an inhibitor of plant PM-ETC, was applied at concentrations ranging from 10 to 100 µM. SHAM (Salicylhydroxamic acid), an inhibitor of alternative oxidase, and myxothiazol, an inhibitor of complex III in mETC, were used in 10 or 100 µM and 10 or 20 µM concentrations, respectively. To observe the possible effect of superoxide on NO elimination, superoxide dismutase (100 U/mL) was added into the reaction mixture.

After these various 30-min long treatments, the root segments were removed, and the fluorescence intensity was measured in 300 µL of the reaction mixture using a microplate reader (SynergyHT BIO-TEK, USA) at Ex/Em: 495 (filter 485/20) / 515 (filter 528/20) nm for DAF-FM and Ex/Em: 552 (filter 560/15) / 578 (filter 590/35) nm for DAR-4 M. The amount of NO generated by root segments was expressed as an increase in relative fluorescence unit (RFU) after the background fluorescence (reaction mixture where root segments were not added for 30 min) was subtracted.

For NO localization, root segments were incubated as described above for NO emission analysis, but instead of cell-impermeable probes, the cell-permeable 25 μM diaminorhodamine-4 M acetoxymethyl ester (DAR-4 M-AM; from 5 mM stock in DMSO) was used. After the incubation, the roots were washed for 5 min with distilled water, and root fluorescence intensity was detected by a fluorescence stereomicroscope (Discovery V12, Zeiss, Germany; Ex/Em: 545 ± 25 / 606 ± 70 nm). The presented images are the representative of five independent experiments. The staining and fluorescence intensity (grey values) were analyzed by the ImageJ software (1.54 k version).

### Extracellular nitrite/nitrate analysis and their uptake by the root tips

Nitrite and nitrate production and their uptake by root segments were monitored fluorimetrically using the Nitrate/Nitrite Fluorometric Assay Kit (Cayman chemicals). Apical 4 mm long segments from barley root tips were washed in 400 µL of 20 mM sodium phosphate buffer, pH 6.0, for 5 min. Depending on the experiment, 15 or 30 segments of roots were used per reaction. After the washing, the root tips were incubated in 300 µL of 20 mM sodium phosphate buffer, pH 6.0, containing 0, 0.25, 0.5, 1, 2, 4, 6 or 8 µM of either nitrite or nitrate for 30 min at 30 °C. After removing the root segments, nitrate was converted to nitrite using nitrate reductase, and the amount of nitrite was then detected by 2,3-diaminonaphthalene according to the manufacturer’s recommendations. The fluorescence signal was recorded with the microplate reader using excitation at 360 (filter 360/40) nm and fluorescence detection at 420 (filter 420/50) nm, and the amount of nitrate/nitrite was expressed as an increase in relative fluorescence unit (RFU).

### Statistical analyses

Each value is the mean of five independent experiments with three replicates per experiment. The data were analyzed by one-way analysis of variance (ANOVA test), and the means were separated using Tukey’s test.

## Results

### Azide, cyanide, DPI and dicumarol increased the accumulation and emission of NO from root tips

**Fig. 1:**
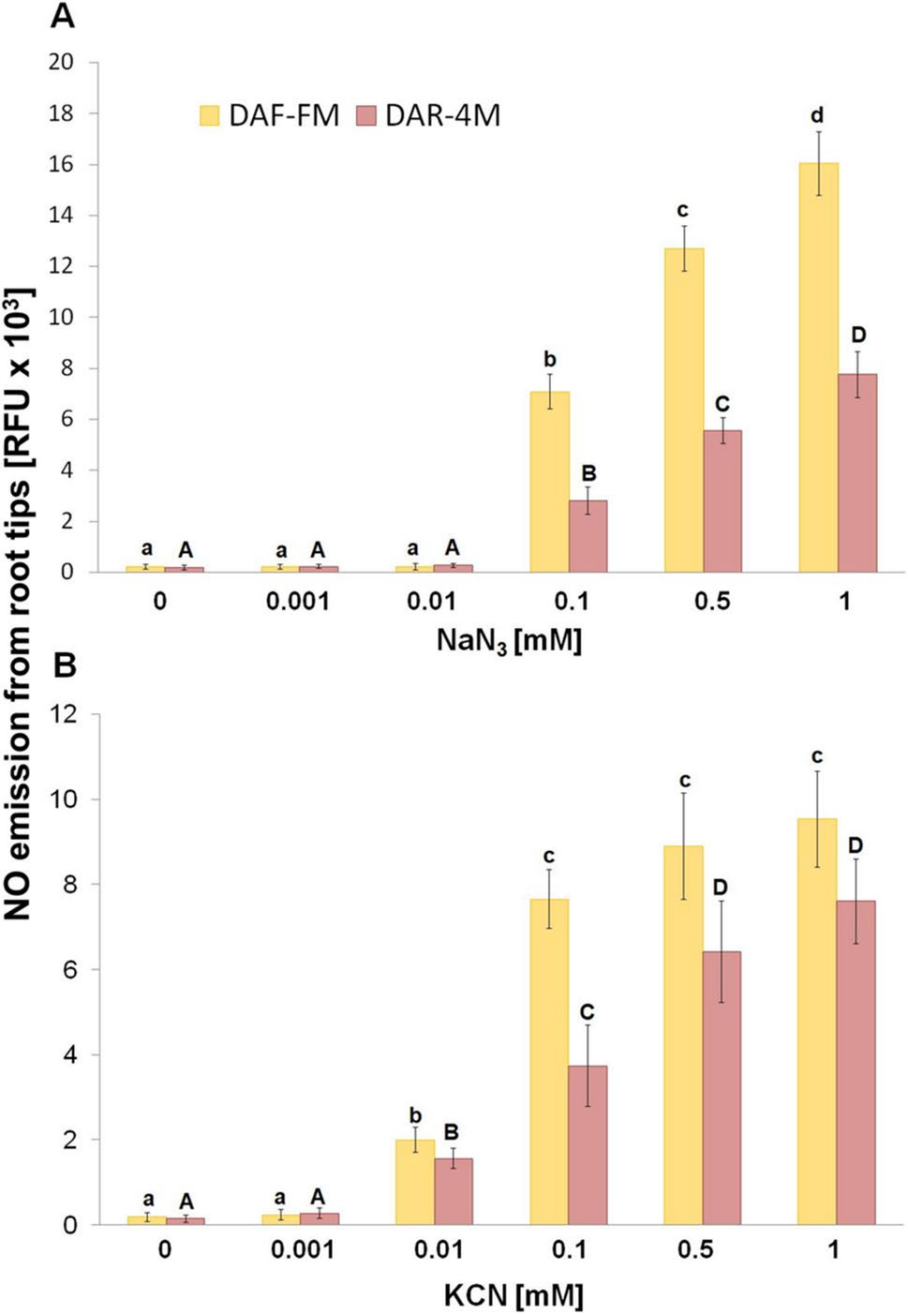
Effect of different concentrations of NaN_3_ **A** and KCN **B** on NO emission from barley root tips into the incubation medium during 30 min of incubation. Mean values ± SD (*n* = 5). Different letters (small letters for DAF-FM and capital letters for DAR-4 M) indicate statistical significance according to Tukey’s test (*P* < *0.05*)

**Fig. 2:**
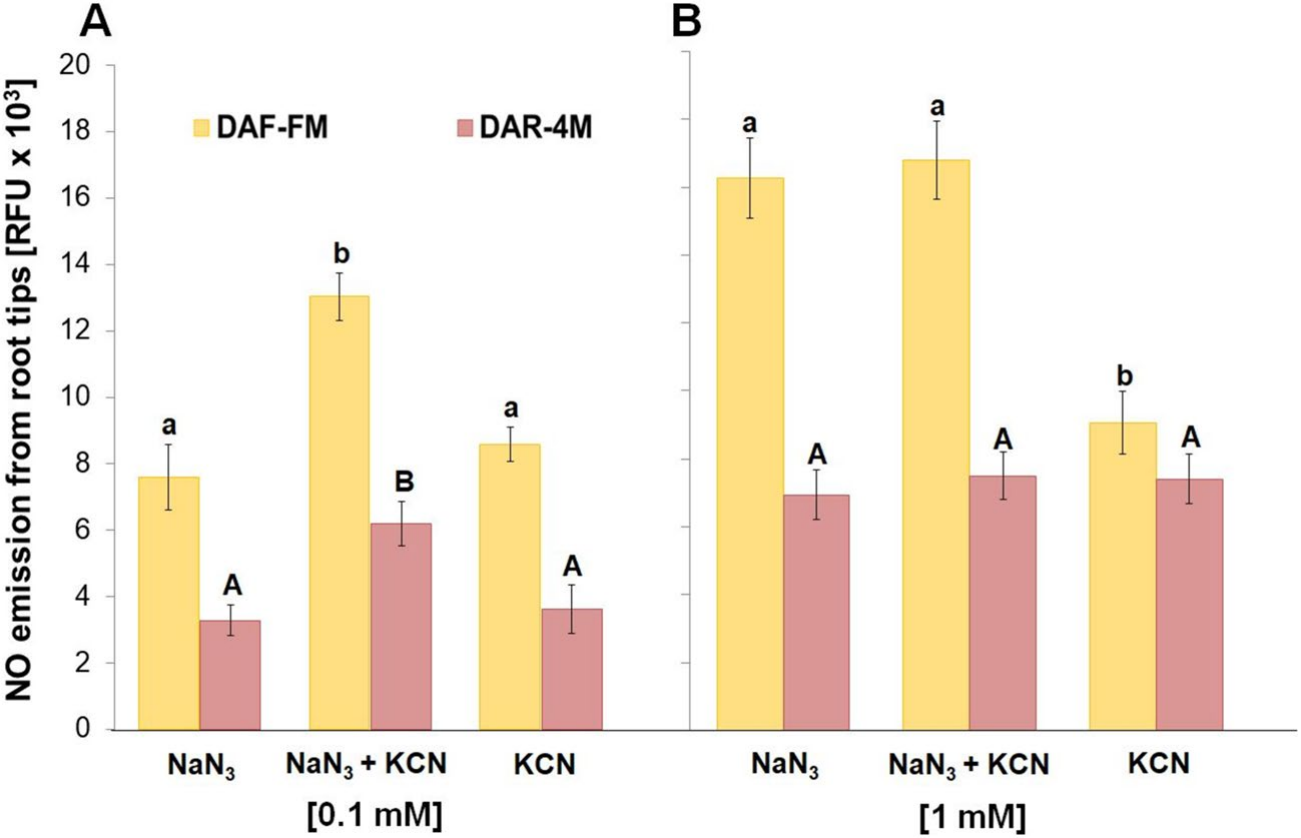
Effect of NaN_3_ and KCN co-treatment at 0.1 **A** and 1 mM **B** concentrations on NO emission from barley root tips into the incubation medium during 30 min of incubation. Mean values ± SD (*n* = 5). Different letters (small letters for DAF-FM and capital letters for DAR-4 M) indicate statistical significance according to Tukey’s test (*P* < *0.05*)

Under our experimental conditions, NO emission from barley root tips into the surrounding medium was only hardly detectable using either DAF-FM or DAR-4 M (Fig. 1). However, the addition of both azide (Fig. 1A) and cyanide (Fig. 1B) markedly increased the amount of NO liberated into the incubation medium, in a concentration-dependent manner. An increase in NO emission was observed already in the presence of 0.1 mM azide in the incubation medium. The measured NO emission was highest at 1 mM azide concentration (Fig. 1A). Cyanide evoked marked NO emission even at 0.01 mM concentration, and increased further with increasing concentrations of cyanide (Fig. 1B). While at lower concentrations the combination of azide and cyanide has an additive effect on the NO emission from root tips, this effect was not observed at higher, 1 mM, concentration (Fig. 2).

**Fig. 3:**
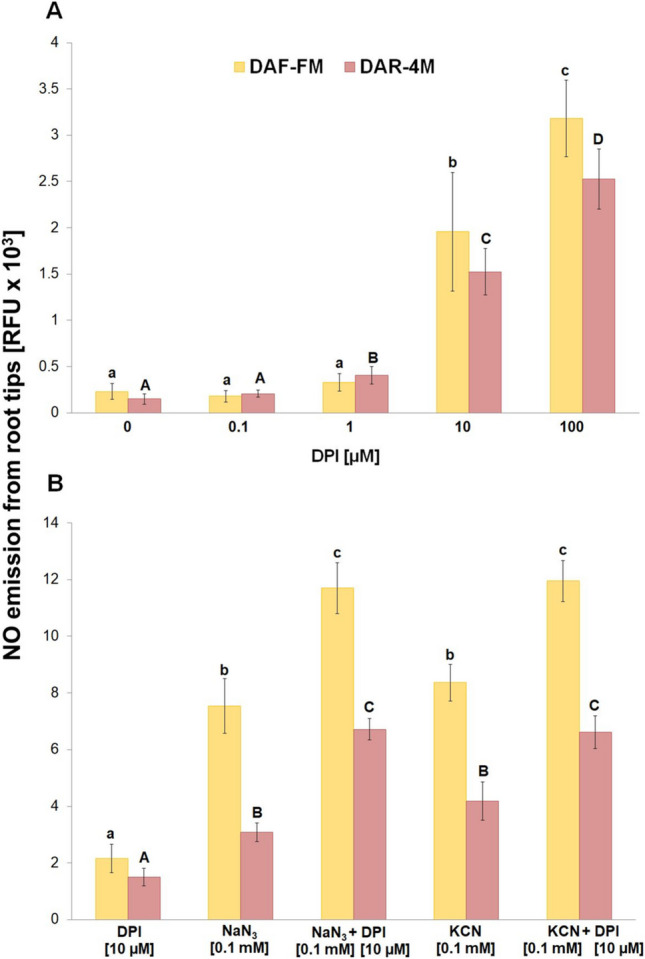
Effect of different concentrations of DPI **A** and co-effect of 10 µM DPI with 0.1 mM NaN_3_ or 10 µM DPI with 0.1 mM KCN **B** on NO emission from barley root tips into the incubation medium during 30 min of incubation. Mean values ± SD (*n* = 5). Different letters (small letters for DAF-FM and capital letters for DAR-4 M) indicate statistical significance according to Tukey’s test (*P* < *0.05*)

**Fig. 4:**
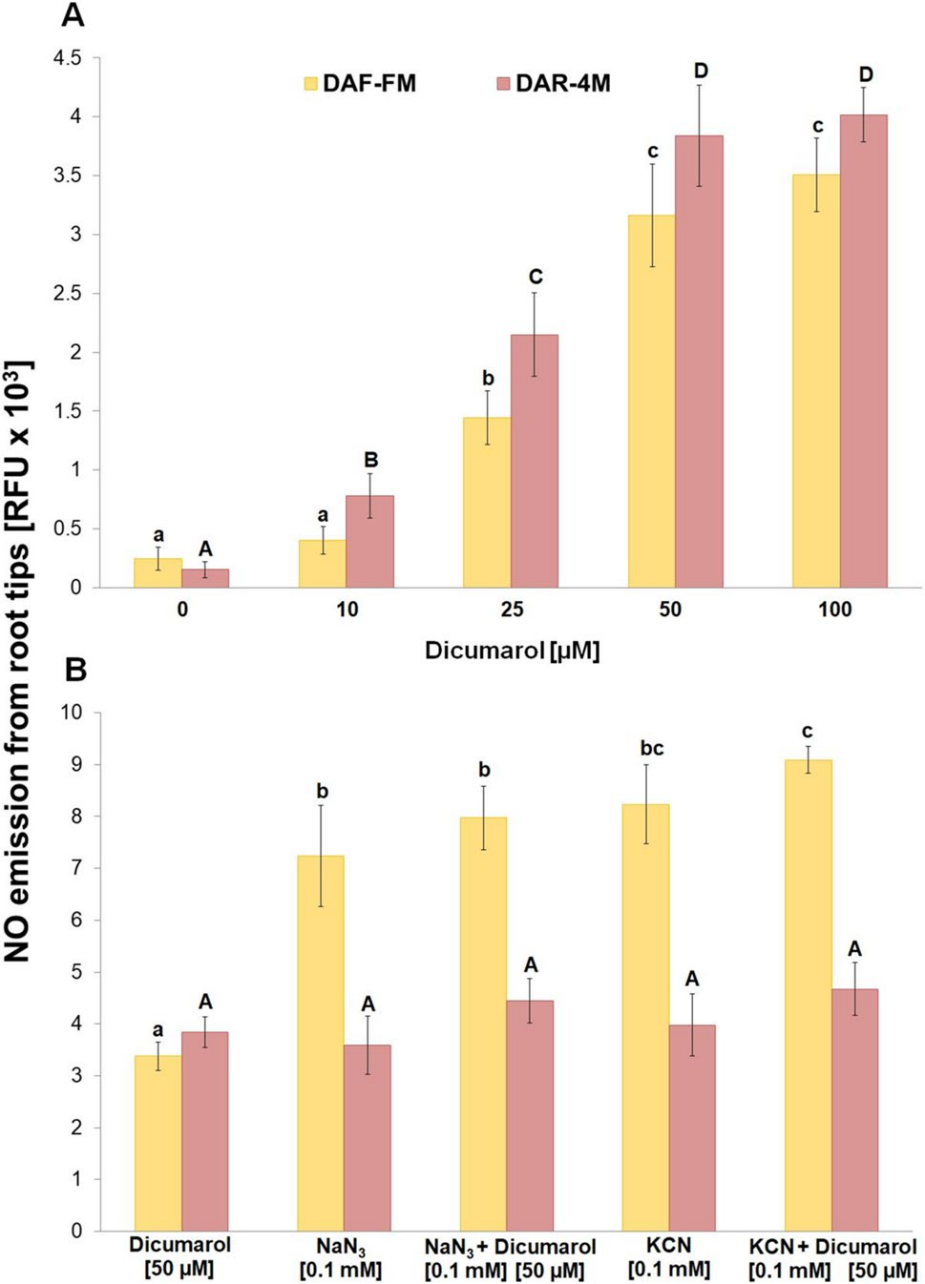
Effect of different concentrations of dicumarol **A** and co-effect of 50 µM dicumarol with 0.1 mM NaN_3_ or 50 µM dicumarol with 0.1 mM KCN **B** on NO emission from barley root tips into the incubation medium during 30 min of incubation. Mean values ± SD (*n* = 5). Different letters (small letters for DAF-FM and capital letters for DAR-4 M) indicate statistical significance according to Tukey’s test (*P* < *0.05*)

DPI increased the emission of NO from root tips into the incubation medium at 10 µM concentration, but it was considerably lower in comparison with azide or cyanide and with increasing concentration of DPI, the detected NO levels increased further (Fig. 3A). In addition, DPI had an additive effect on the amount of released NO in combination with both azide and cyanide (Fig. 3B). An increased level of NO in the incubation medium was also observed in the presence of dicumarol (Fig. 4A). This increase started at 10 µM and reached the maximum level at 50 µM concentration of dicumarol. However, in contrast to DPI, it had no additive effect either with azide or cyanide on NO emission from the root tips (Fig. 4B). The DMSO in the reaction mixture at the used concentrations in our experiments had no effect on the DAF-FM or DAR-4 M fluorescence signal either without or with azide or cyanide (Suppl. 1).

**Fig. 5:**
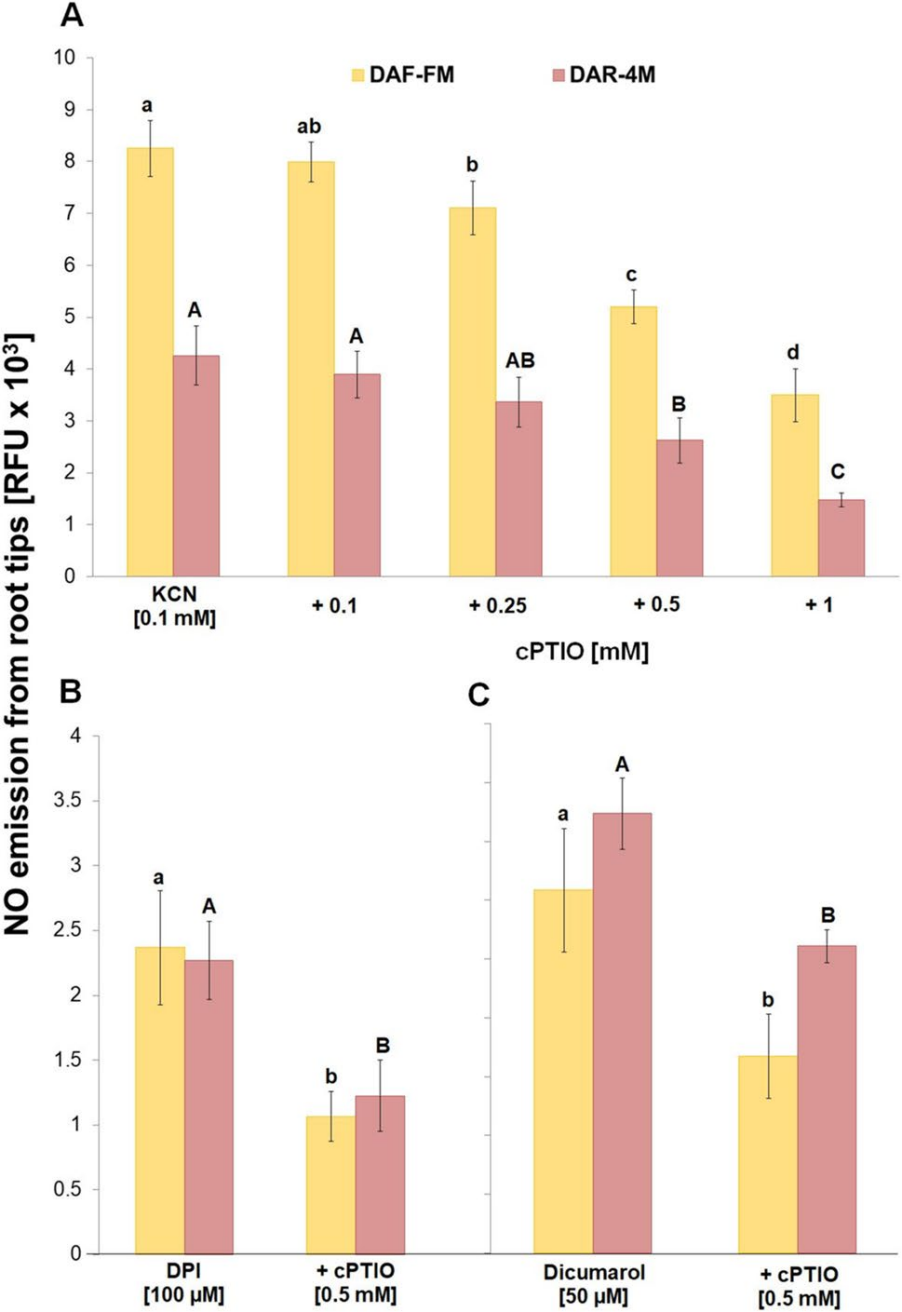
Co-effect of 0.1 mM KCN with different concentrations of cPTIO **A**, co-effect of 100 µM DPI with 0.5 mM cPTIO **B** and co-effect of 50 µM dicumarol with 0.5 mM cPTIO (**C**) on NO emission from barley root tips into the incubation medium during 30 min of incubation. Mean values ± SD (*n* = 5). Different letters (small letters for DAF-FM and capital letters for DAR-4 M) indicate statistical significance according to Tukey’s test (*P* < *0.05*)

The addition of cPTIO into the incubation medium markedly decreased the amount of detectable NO in the presence of cyanide in a dose-dependent manner (Fig. 5A), and this effect was also observed in the case of DPI or dicumarol (Fig. 5B, C). At the same time, the combination of azide, cPTIO and either DAF-FM or DAR-4 M in the incubation medium without root tips led to a false positive fluorescence in a cPTIO concentration-dependent manner (Suppl. 2). By contrast, the presence of high superoxide dismutase activity in the incubation medium did not affect the amount of released NO by the root tips (Suppl. 3).

**Fig. 6:**
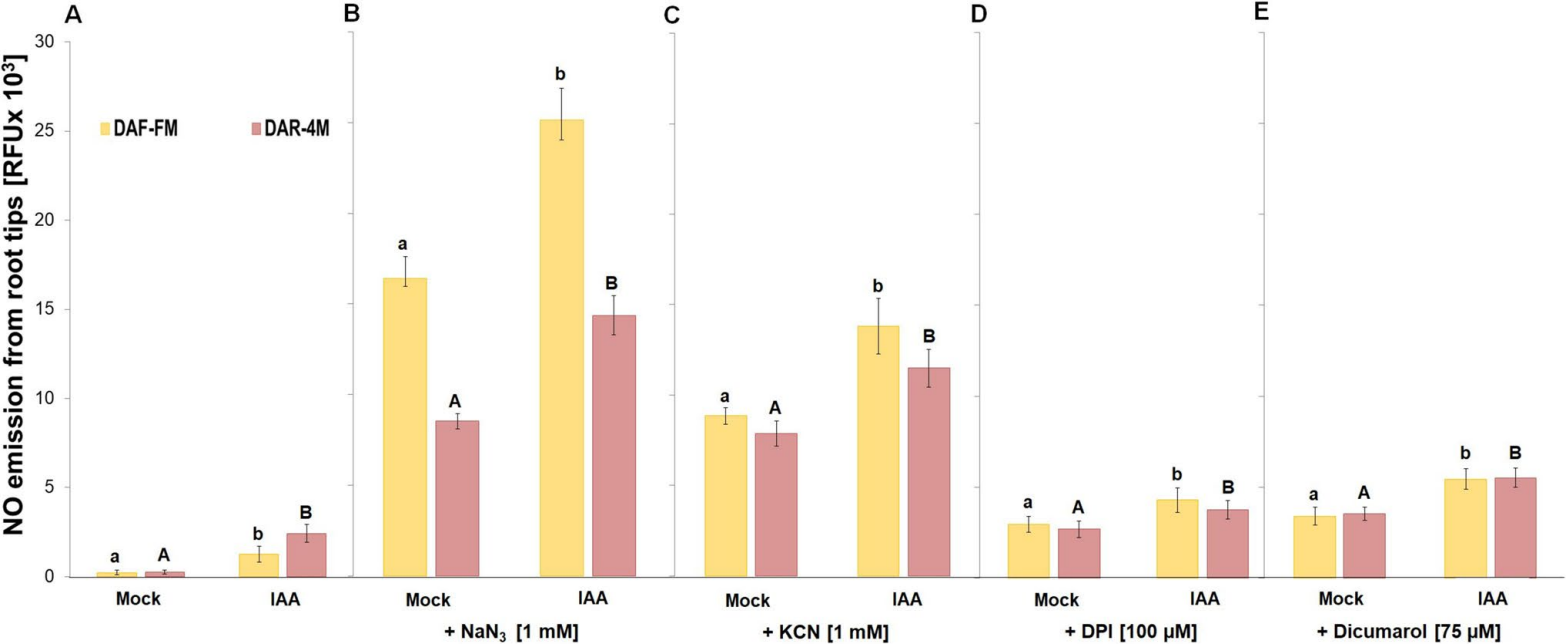
Effect of IAA on NO emission **A** and the effect of 1 mM NaN_3_ **B**, 1 mM KCN **C**, 100 µM DPI **D** or 75 µM dicumarol **E** on IAA-induced NO emission from barley root tips into the incubation medium during 30 min of incubation. Mean values ± SD (*n* = 5). Different letters (small letters for DAF-FM and capital letters for DAR-4 M) indicate statistical significance according to Tukey’s test (*P* < *0.05*)

**Fig. 7:**
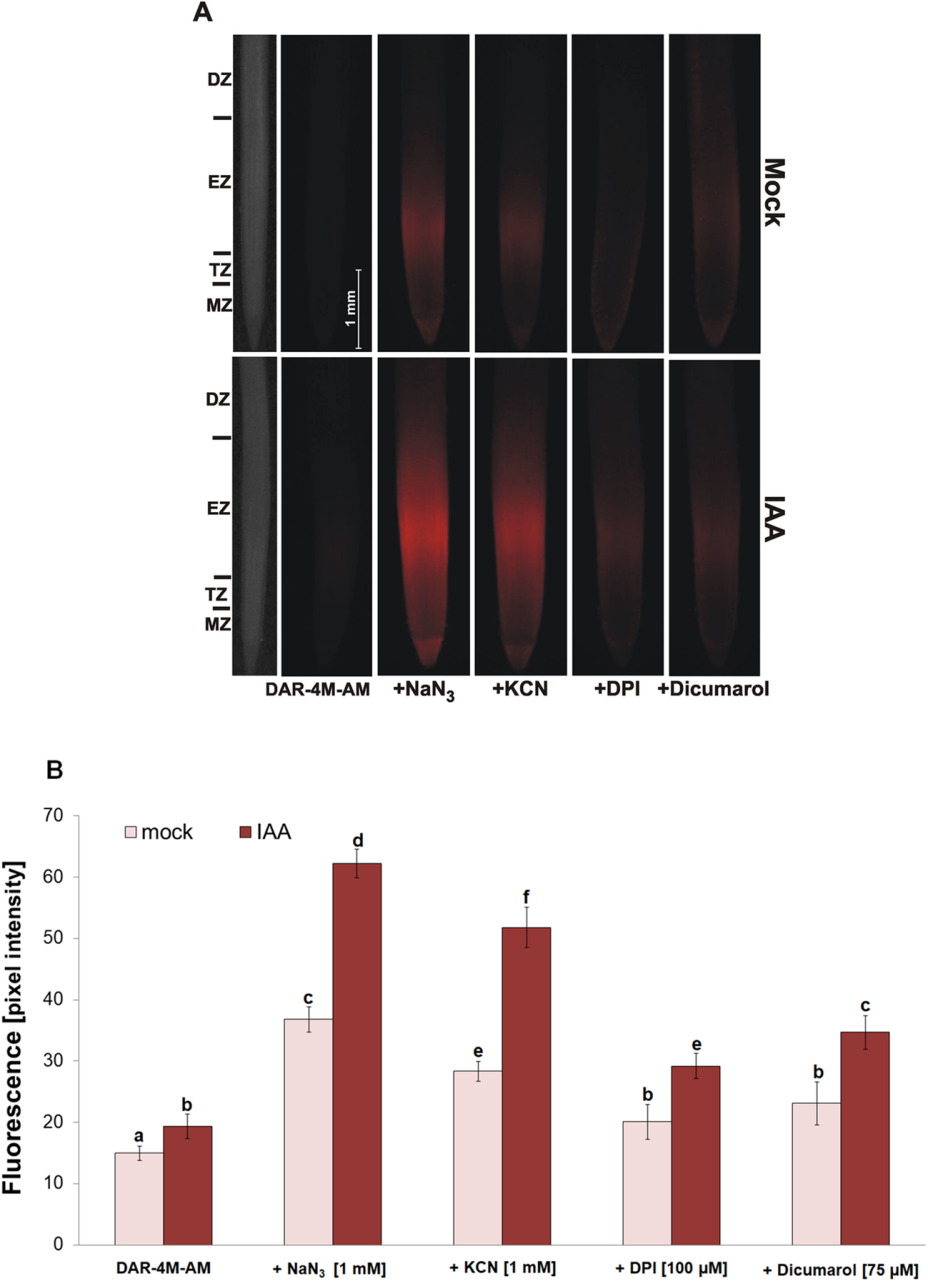
Effect of 1 mM NaN_3_, 1 mM KCN, 100 µM DPI and 75 µM dicumarol in staining solution on NO accumulation in root tips **A** and on fluorescence intensity **B** 3 h after 0 (Mock) and 10 µM IAA-treatment. MZ, TZ, EZ and DZ show meristematic, transition, elongation and differentiation zone of root tip. Mean values ± SD (*n* = 5). Different letters indicate statistical significance according to Tukey’s test (*P* < *0.05*)

In IAA-treated roots, significantly increased NO emission was detected from the root tips 3 h after short-term treatment in comparison with untreated roots (Fig. 6A). This IAA-induced increased NO production was observed also in the presence of azide and cyanide; however, it was several times higher in comparison with experiments without the addition of azide or cyanide (Fig. 6B, C). Similarly, this increased NO production was detectable in IAA-treated root tips in the presence of DPI and dicumarol (Fig. 6D, E). Using a cell-permeable probe (DAR-4 M-AM), we showed that the level of NO in the presence of azide, cyanide, DPI or dicumarol increased not only in the incubation medium but also inside the cells of both untreated (Fig. 7A) and IAA-treated roots (Fig. 7B). This signal was localized mainly into root transition and elongation zones (Fig. 7A, B).

### Root tips effectively consume a large amount of externally applied NO

**Fig. 8:**
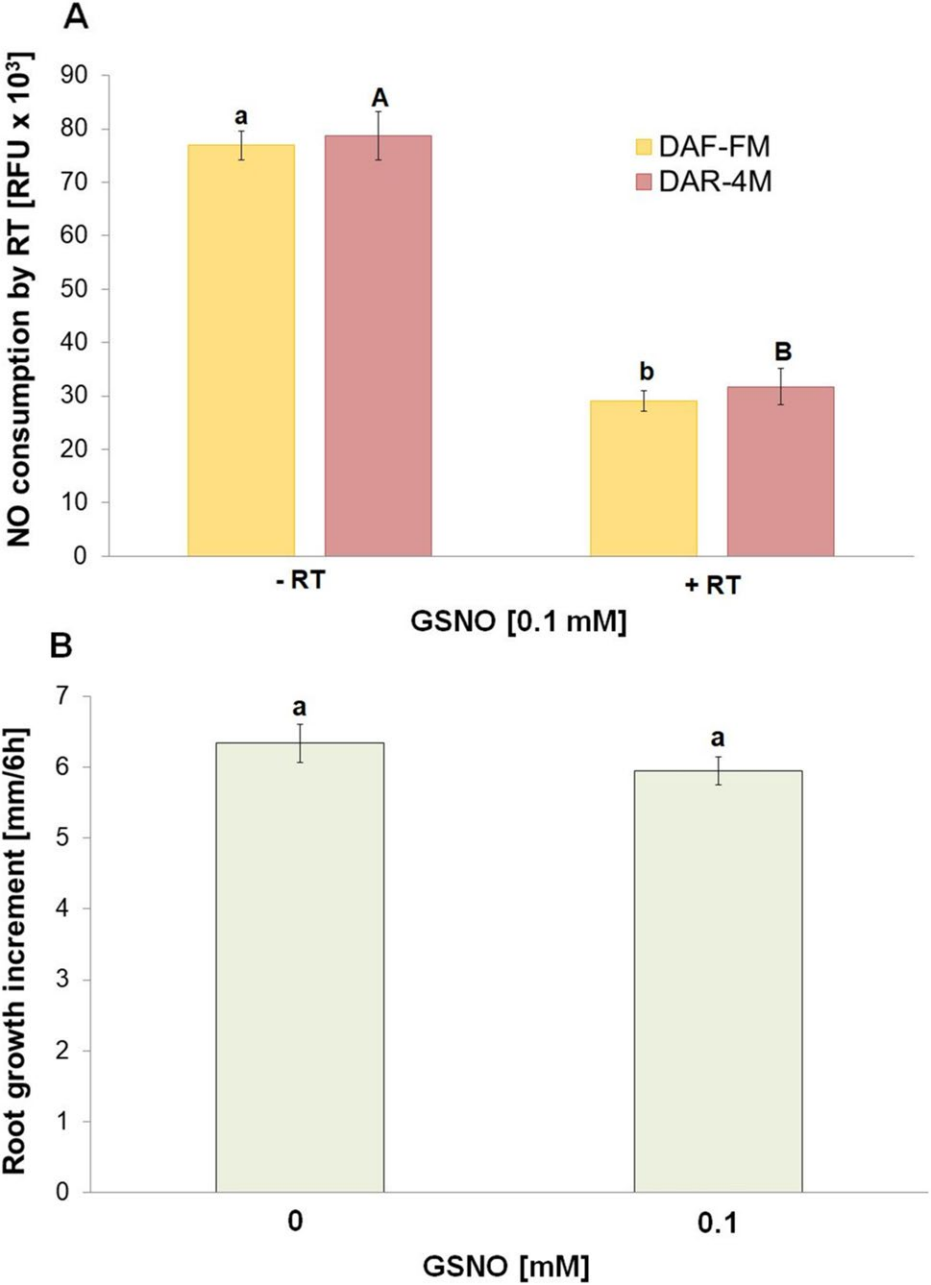
Effect of root tips (RT) on the NO level released from NO donor GSNO in the incubation medium **A** and root growth increment 6 h after the short-term (30 min) treatment of roots of intact seedlings with 0.1 mM GSNO. **B**. Mean values ± SD (*n* = 5). Different letters (small letters for DAF-FM and capital letters for DAR-4 M) indicate statistical significance according to Tukey’s test (*P* < *0.05*)

The addition of NO donors, GSNO, into the incubation medium revealed that root tips decomposed a large amount of NO liberated from GSNO (Fig. 8A). Moreover, the root growth analysis showed that intact roots can withstand the very high concentrations of externally applied NO during this short-term incubation without marked reduction in their growth (Fig. 8B). However, in the presence of higher, 1 mM, GSNO concentration a marked root growth inhibition was detected in comparison with control roots (control root growth increment: 6.34 ± 0.27 mm / 6 h; after treatment with 1 mM GSNO for 30 min: 3.88 ± 0.39 mm / 6 h).

We also analyzed the nitrite and nitrate levels in the incubation medium, as potential metabolic products of NO after the decomposition of NO by root tips. However, we were not able to detect their levels after the 30-min incubation period (data not shown). On the other hand, the root tips extremely rapidly took up the externally applied nitrite or nitrate from the incubation medium (Suppl. 4). However, this externally applied nitrate or nitrite into the incubation medium had no effect on NO emission from the root tips (Suppl. 5).

### SHAM and myxothiazol do not influence the emission of NO from root tips

**Fig. 9:**
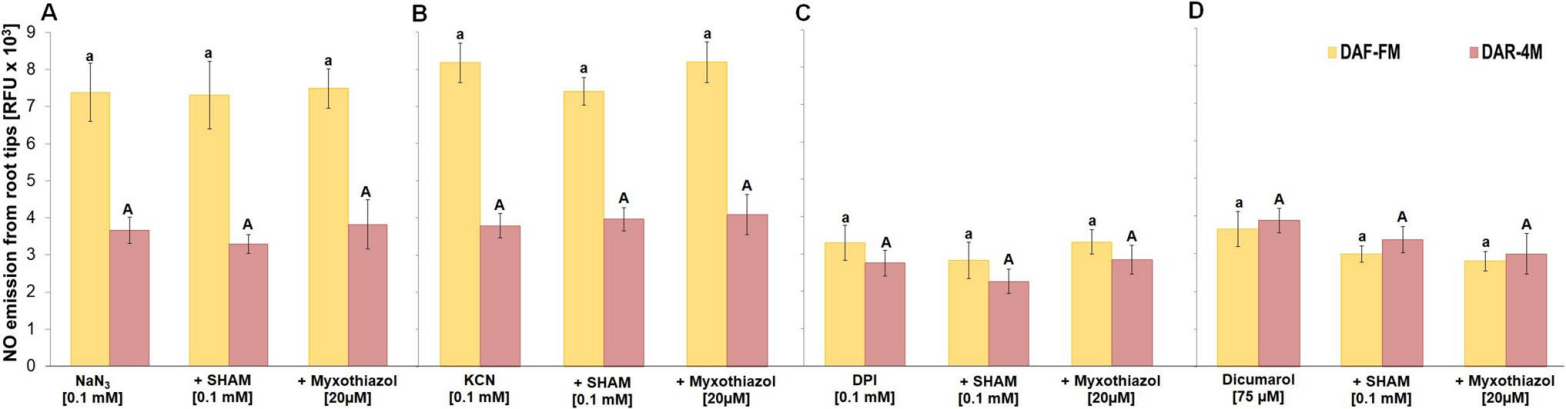
Effect of 100 µM SHAM and 20 µM myxothiazole on NO emission from barley root tips into the incubation medium during 30 min of incubation in the presence of 0.1 mM NaN_3_ **A**, 0.1 mM KCN **B**, 0.1 mM DPI **C** and 75 µM dicumarol **D**. Mean values ± SD (*n* = 5). Different letters (small letters for DAF-FM and capital letters for DAR-4 M) indicate statistical significance according to Tukey’s test (*P* < *0.05*)

During the short-term treatment, SHAM or myxothiazol did not evoke a significant change in NO emission from the root tips (Suppl. 6). In addition, they did not significantly affect the azide-, cyanide-, DPI- or dicumarol-evoked enhanced NO emission from barley root tips into the incubation medium (Fig. 9).

## Discussion

Earlier studies have demonstrated that NO emission, to various degrees, is possibly a common feature for all plant species (Rockel et al. 1996; Wildt et al. 1997). In leaf, NO emission exhibited large variation between different plant species, and this variation showed a negative correlation with leaf nitrogen level and net photosynthetic rate (Chen et al. 2015). In roots, NO in the apoplast may function in nitrate sensing and metabolism, but its role in the defense against pathogens and abiotic stresses have also been suggested (Stöhr and Ullrich 2002).

During our experimental conditions, NO emission from barley root tips was hardly detectable. However, after the addition of flavohemeprotein inhibitors such as azide, cyanide or DPI and an inhibitor of plasma membrane electron transport, dicumarol (Döring et al. 1992), a marked increase in both the level of NO in root tips and the amount of NO released into the surrounding medium was observed. These results suggest that the majority of NO is rapidly consumed by flavohemeproteins in roots, and that is a possible reason why a much lower level of NO is detectable in the root cells and in the incubation medium during control conditions. In addition, externally applied NO, released from the NO donor GSNO, rapidly degraded in the presence of root tip cells without affecting root growth, indicating that root tips are capable of catabolizing a much higher amount of NO than produced by root tip cells themselves under physiological conditions.

It is well documented that NO is a physiological substrate for both plant and mammalian peroxidases, leading to nitrite formation through the nitrosonium cation (Glover et al. 1999; Abu-Soud and Hazen 2000). The application of azide, a peroxidase inhibitor, strongly inhibited the NO consumption by mammalian myeloperoxidases (Abu-Soud and Hazen 2000). Likewise, azide strongly inhibited the consumption of NO by barley root tips, leading to the NO accumulation in the cells of root tips and also to the enhanced emission of NO into the incubation medium.

Flavohemoglobins are widespread among bacteria and yeasts and are involved in NO scavenging either through its reduction under anaerobic conditions to nitrous oxide or through its oxidation to nitrate in the presence of oxygen (Bonamore and Boffi 2008). Our results showed that DPI, a well-known inhibitor of flavoproteins, had an inhibitory effect on NO consumption in the cells of barley root tips. In line with this observation, it has been previously demonstrated that alfalfa root cultures expressing barley hemoglobin for the effective metabolism of NO to nitrate via NOD-like activity require DPI-sensitive flavoproteins utilizing NAD(P)H as the electron donor (Igamberdiev et al. 2004). Moreover, the NOD activity of prokaryotic flavohemoglobins was sensitive to cyanide (Gardner et al. 1998). In the same manner, cyanide, even at low concentrations considerably inhibited extracellular NO catabolism in the barley root tips. It has been previously reported that cyanide affects wheat root growth by enhancing NO levels, probably by inhibiting NO detoxifying systems (Groppa et al. 2008). In line with these observations in plants, in several mammalian cell lines, robust NO consumption activity was observed, which was oxygen-dependent and strongly inhibited by DPI, azide and cyanide, indicating that this NOD activity is catalyzed by flavin- and heme-containing proteins (Gardner et al. 2001; Hallstrom et al. 2004; Schmidt and Mayer 2004). In addition, the NO inactivation was associated with membranes, and this activity is lost upon cell disruption, suggesting that intact vital cells are required for NO catabolic activity. Our results also demonstrated that besides flavohemeprotein inhibitors the inhibition of plasma membrane electron transport by dicumarol also lowered the NO consumption in barley root tips. These findings suggest that the plasma membrane-associated flavohemeproteins are probably responsible for the NO consumption activity of root tip cells.

On the other hand, the flavohemeprotein inhibitors at concentrations at which they inhibited NO consumption had no effect on NO synthesis in barley root tips, which led to its enhanced emission into the surroundings of root tips and also to its accumulation inside the root cells. In agreement with these results, it has been previously shown that NO formation in the purified plasma membrane of tobacco roots occurred via nitrite reduction, and this nitrite-reducing enzyme activity was insensitive to cyanide (Stöhr et al. 2001). It is well documented in the roots of higher plants that the plasma membrane-bound NR reduces apoplastic nitrate with both NADH and succinate as electron donors (Stöhr and Ullrich 1997). This apoplastically generated nitrite is rapidly converted to NO by plasma membrane-bound nitrite-NO reductase (PM-NI-NOR) using the same electron donors as PM-NR, probably via phylloquinone (Stöhr et al. 2001; Eick and Stöhr 2012). While azide inhibited plasma membrane NR-mediated nitrate reduction in isolated plasma membrane vesicles from soybean roots (Eick and Stöhr 2012), under our experimental conditions, it markedly increased NO emission in vivo from barley roots. Therefore, in barley roots, the nitrate reduction activity of NR is probably not directly involved in NO generation. It has been shown recently, that NR can regulate NO level indirectly by supplying electrons from NAD(P)H to other redox proteins, including truncated hemoglobins with dioxygenase activity and molybdoenzymes with reductase activity (Chamizo-Ampudia et al. 2017).

In NR- and nitrite reductase-deficient green alga cells grown on nitrite as substrate, the NO production was inhibited by cyanide and inhibitors of mitochondrial complex III, suggesting that the nitrite reduction to NO takes place via the mETC (Tischner et al. 2004). In higher plants, root mitochondria are capable of nitrite reduction to NO, which can be blocked by myxothiazol and SHAM, an inhibitor of mETC at complex III and alternative oxidase, respectively (Gupta and Igamberdiev 2011; Igamberdiev et al. 2010). In addition to myxothiazol and SHAM, cyanide also inhibited the emission of NO from both tobacco cell suspensions and isolated mitochondria, suggesting that mETC was the major source of NO emission via nitrite reduction (Planchet et al. 2005). Similarly, in isolated barley and rice mitochondria, NO generation was inhibited by mETC inhibitors, such as DPI, myxothiazol and cyanide (Stoimenova et al. 2007). On the contrary, in barley root tips, azide, cyanide and DPI had a considerable stimulating effect within a very short period on the NO emission. This effect was not influenced by the presence of myxothiazol or SHAM, suggesting that mETC is not involved in NO accumulation and emission from barley root tips. On the other hand, when PM-ETC, which transports electrons from electron donors to several plasma membrane flavohemeproteins, was inhibited by dicumarol, an enhanced NO emission was observed. Since dicumarol, similarly to azide, cyanide and DPI, enhanced the NO emission from the root tip, there may be a probability that the plasma membrane redox system is involved in the consumption of NO by root tip cells. In addition, externally applied nitrite/nitrate, a substrate for mitochondrial-driven NO synthesis, did not increase NO emission from the root tips during this short incubation time.

In water fern, azide-dependent NO emission was observed; however, in contrast to our result, where an additive effect was observed between azide and cyanide on NO emission, NO production in water fern was strongly inhibited by cyanide (Gurung et al. 2014). This NO production from azide is inhibited by 3-aminotriazole, suggesting that this process is catalyzed by a catalase-like activity.

Whereas several reports showed a rapid turnover of extracellular NO both in animal and plant cells, including our experiments, the function of this rapid NO metabolism is only poorly explained. Myeloperoxidases modulate the vascular signaling and vasodilatory function of NO in humans during acute inflammation by converting it to nitrite (Eiserich et al. 2002). The observation that roots of several plant species, including barley, exhibited enhanced NO emission under low oxygen conditions (Chen et al. 2010; Liu et al. 2015; Welle et al. 2024) may be associated with the inhibition of NO catabolism by dioxygenases, which requires oxygen. Seed-specific hemoglobin in sugar beet, BvHb1.2, exhibited high NOD activity, and the authors suggest that during seed germination, it has a crucial role in nitrate synthesis when the external nitrate supply is limited and this way, a sufficient amount of nitrite is generated from NO and oxygen during seed germination (Eriksson et al. 2019).

The high NO-consuming activity of barley root tips efficiently degraded even the externally applied high concentrations of NO without marked root growth inhibition. However, at very high GSNO concentration the amount of liberated NO is toxic even during this short exposure time, evoking root growth inhibition. These results suggest that this high rate of NO catabolic activity of root tip cells is probably required for a normal root performance and is involved in the regulation of NO level in the root tips resulting in the detoxification of superfluous NO.

## Conclusion

**Fig. 10:**
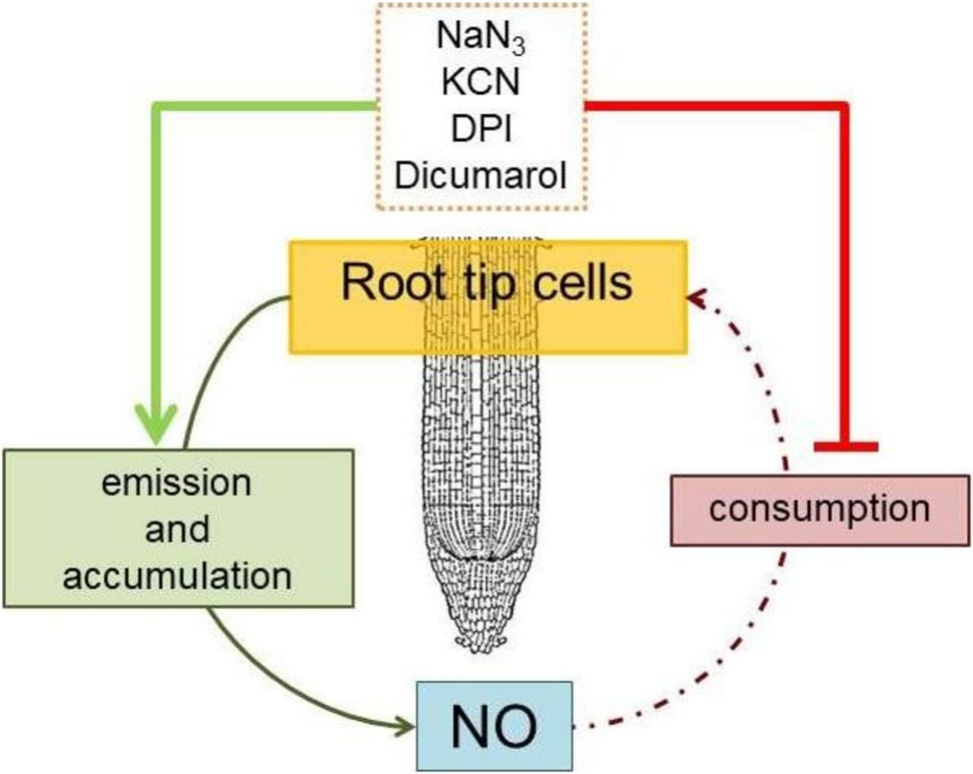
Graphical summary of inhibitors affecting NO emission and consumption in barley root tips

Based on these results, it can be concluded that barley root tips generate a considerable amount of NO; however, almost the entire amount of NO is rapidly consumed by flavohemeproteins (Fig. 10). Application of flavohemeprotein inhibitors, such as azide, cyanide, DPI and dicumarol, an inhibitor of the plasma membrane electron transport chain, inhibited the NO consumption by root tip cells, resulting in both NO accumulation in cells and enhanced NO emission from root tips. These results suggest that the NO consumption activity of root tip cells plays an important role in the regulation of NO level in barley root tips.

## Supplementary Information

Below is the link to the electronic supplementary material.

Supplementary file1 (JPG 63 KB)

Supplementary file2 (JPG 48 KB)

Supplementary file3 (JPG 89 KB)

Supplementary file4 (JPG 89 KB)

Supplementary file5 (JPG 55 KB)

Supplementary file6 (JPG 39 KB)

## Data Availability

All data generated or analyzed during this study are included in this published article.

## References

[CR1] Abu-Soud HM, Hazen SL (2000) Nitric oxide is a physiological substrate for mammalian peroxidases. J Biol Chem 275:37524–37532. 10.1074/jbc.275.48.37524 11090610 10.1074/jbc.275.48.37524

[CR2] Amdahl MB, DeMartino AW, Gladwin MT (2020) Inorganic nitrite bioactivation and role in physiological signaling and therapeutics. Biol Chem 401:201–211. 10.1515/hsz-2019-0349 10.1515/hsz-2019-0349 31747370

[CR3] Arasimowicz-Jelonek M, Floryszak-Wieczorek J (2011) Understanding the fate of peroxynitrite in plant cells – From physiology to pathophysiology. Phytochem 72:681–688. 10.1016/j.phytochem.2011.02.025 10.1016/j.phytochem.2011.02.025 21429536

[CR4] Astier J, Gross I, Durner J (2018) Nitric oxide production in plants: an update. J Exp Bot 69:3401–3411. 10.1093/jxb/erx420 29240949 10.1093/jxb/erx420

[CR5] Bender D, Schwarz G (2018) Nitrite-dependent nitric oxide synthesis by molybdenum enzymes. FEBS L 592:2126–2139. 10.1002/1873-3468.13089 10.1002/1873-3468.13089 29749013

[CR6] Bethke PC, Badger MR, Jones RL (2004) Apoplastic synthesis of nitric oxide by plant tissues. Plant Cell 16:332–341. 10.1105/tpc.017822 14742874 10.1105/tpc.017822 PMC341907

[CR7] Bonamore A, Boffi A (2008) Flavohemoglobin: Structure and reactivity. IUBMB Life 60:19–28. 10.1002/iub.9 18379989 10.1002/iub.9

[CR8] Broniowska KA, Diers AR, Hogg N (2013) S-nitrosoglutathione. Biochim Biophys Acta 1830:3173–3181. 10.1016/j.bbagen.2013.02.004 23416062 10.1016/j.bbagen.2013.02.004 PMC3679660

[CR9] Chamizo-Ampudia A, Sanz-Luque E, Llamas A, Galvan A, Fernandez E (2017) Nitrate reductase regulates plant nitric oxide homeostasis. Trends Plant Sci 22:163–174. 10.1016/j.tplants.2016.12.001 28065651 10.1016/j.tplants.2016.12.001

[CR10] Chen J, Xiao Q, Wu FH, Pei ZM, Wang J, Wu YG, Zheng HL (2010) Nitric oxide emission from barley seedlings and detached leaves and roots treated with nitrate and nitrite. Plant Soil Environ 56:201–208. 10.17221/231/2009-pse

[CR11] Chen J, Wang C, Wu F-H, Wang W-H, Liu T-W, Chen J, Xiao Q, He B-Y, Zheng H-L (2015) Variation of nitric oxide emission potential in plants: a possible link to leaf N content and net photosynthetic activity. J Plant Ecol 8:313–320. 10.1093/jpe/rtu015

[CR12] Coffey MJ, Natarajan R, Chumley PH, Coles B, Thimmalapura P-R, Nowell M, Kühn H, Lewis MJ, Freeman BA, O`Donnell VB, (2001) Catalytic consumption of nitric oxide by 12/15-lipoxygenase: Inhibition of monocyte soluble guanylate cyclase activation. Proc Natl Acad Sci 98:8006–8011. 10.1073/pnas.141136098 11427723 10.1073/pnas.141136098 PMC35458

[CR13] Cooney RV, Harwood PJ, Custer LJ, Franke AA (1994) Light-mediated conversion of nitrogen dioxide to nitric oxide by carotenoids. Environ Health Perspect 102:460–462. 10.1289/ehp.94102460 8593849 10.1289/ehp.94102460 PMC1567141

[CR14] Corpas FJ, Barroso JB, Carreras A, Quirós M, León AM, Romero-Puertas MC, Esteban FJ, Valderrama R, Palma JM, Sandalio LM, Gómez M, del Río LA (2004) Cellular and subcellular localization of endogenous nitric oxide in young and senescent pea plants. Plant Physiol 136:2722–2733. 10.1104/pp.104.042812 15347796 10.1104/pp.104.042812 PMC523336

[CR15] Corpas FJ, Barroso JB, Carreras A, Valderrama R, Palma JM, León AM, Sandalio LM, del Río LA (2006) Constitutive arginine-dependent nitric oxide synthase activity in different organs of pea seedlings during plant development. Planta 224:246–254. 10.1007/s00425-005-0205-9 16397797 10.1007/s00425-005-0205-9

[CR16] Corpas FJ, Palma JM, del Río LA, Barroso JB (2009) Evidence supporting the existence of L-arginine-dependent nitric oxide synthase activity in plants. New Phytol 184:9–14. 10.1111/j.1469-8137.2009.02989.x 19659743 10.1111/j.1469-8137.2009.02989.x

[CR17] Corpas FJ, Alché JD, Barroso JB (2013) Current overview of S-nitrosoglutathione (GSNO) in higher plants. Front Plant Sci 4:126. 10.3389/fpls.2013.00126 23658557 10.3389/fpls.2013.00126 PMC3647110

[CR18] Desikan R, Griffiths R, Hancock J, Neill S (2002) A new role for an old enzyme: Nitrate reductase-mediated nitric oxide generation is required for abscisic acid-induced stomatal closure in *Arabidopsis thaliana*. Proc Natl Acad Sci 99:16314–16318. 10.1073/pnas.252461999 12446847 10.1073/pnas.252461999 PMC138608

[CR19] Domingos P, Prado AM, Wong A, Gehring C, Feijo JA (2015) Nitric oxide: A multitasked signaling gas in plants. Mol Plant 8:506–520. 10.1016/j.molp.2014.12.010 25680232 10.1016/j.molp.2014.12.010

[CR20] Döring O, Lüthje S, Böttger M (1992) Inhibitors of the plasma membrane redox system of *Zea mays* L. roots. The vitamin K antagonists dicumarol and warfarin. Biochim Biophys Acta 1110:235–238. 10.1016/0005-2736(92)90364-R 1382600 10.1016/0005-2736(92)90364-r

[CR21] Eick M, Stöhr C (2012) Denitrification by plant roots? New aspects of plant plasma membrane-bound nitrate reductase. Protoplasma 249:909–918. 10.1007/s00709-011-0355-5 22160216 10.1007/s00709-011-0355-5

[CR22] Eiserich JP, Baldus S, Brennan M-L, Ma W, Zhang C, Tousson A, Castro L, Lusis AJ, Nauseef WM, White CR, Freeman BA (2002) Myeloperoxidase, a leukocyte-derived vascular NO oxidase. Science 296:2391–2394. 10.1126/science.1106830 12089442 10.1126/science.1106830

[CR23] Eriksson NL, Reeder BJ, Wilson MT, Bülow L (2019) Sugar beet hemoglobins: reactions with nitric oxide and nitrite reveal differential roles for nitrogen metabolism. Biochem J 476:2111–2125. 10.1042/BCJ20190154 31285352 10.1042/BCJ20190154 PMC6668756

[CR24] Frungillo L, Skelly MJ, Loake GJ, Spoel SH, Salgado I (2014) S-nitrosothiols regulate nitric oxide production and storage in plants through the nitrogen assimilation pathway. Nat Commun 5:5401. 10.1038/ncomms6401 25384398 10.1038/ncomms6401 PMC4229994

[CR25] Gardner PR, Gardner AM, Martin LA, Salzman AL (1998) Nitric oxide dioxygenase: An enzymic function for flavohemoglobin. Proc Natl Acad Sci USA 95:10378–10383. 10.1073/pnas.95.18.10378 9724711 10.1073/pnas.95.18.10378 PMC27902

[CR26] Gardner PR, Martin LA, Hall D, Gardner AM (2001) Dioxygen-dependent metabolism of nitric oxide in mammalian cells. Free Rad Biol Med 31:191–204. 10.1016/s0891-5849(01)00569-x 11440831 10.1016/s0891-5849(01)00569-x

[CR27] Glover RE, Koshkin V, Dunford HB, Mason RP (1999) The reaction rates of NO with horseradish peroxidase compounds I and II. Nitric Oxide 3:439–444. 10.1006/niox.1999.0256 10637121 10.1006/niox.1999.0256

[CR28] Groppa MD, Rosales EP, Iannone MF, Benavides MP (2008) Nitric oxide, polyamines and Cd-induced phytotoxicity in wheat roots. Phytochem 69:2609–2615. 10.1016/j.phytochem.2008.07.016 10.1016/j.phytochem.2008.07.016 18789805

[CR29] Gupta KJ, Igamberdiev AU (2011) The anoxic plant mitochondrion as a nitrite: NO reductase. Mitochondrion 11:537–543. 10.1016/j.mito.2011.03.005 21406251 10.1016/j.mito.2011.03.005

[CR30] Gurung S, Cohen MF, Yamasaki H (2014) Azide-dependent nitric oxide emission from the water fern *Azolla pinnata*. Russ J Plant Physiol 61:543–547. 10.1134/S1021443714040086

[CR31] Hallstrom CK, Gardner AM, Gardner PR (2004) Nitric oxide metabolism in mammalian cells: Substrate and inhibitor profiles of a NADPH-cytochrome P450 oxidoreductase-coupled microsomal nitric oxide dioxygenase. Free Rad Biol Med 37:216–228. 10.1016/j.freeradbiomed.2004.04.031 15203193 10.1016/j.freeradbiomed.2004.04.031

[CR32] Hebelstrup KH, Igamberdiev AU, Hill RD (2007) Metabolic effects of hemoglobin gene expression in plants. Gene 398:86–93. 10.1016/j.gene.2007.01.039 17555891 10.1016/j.gene.2007.01.039

[CR33] Igamberdiev AU, Seregélyes C, Manac’h N, Hill RD (2004) NADH-dependent metabolism of nitric oxide in alfalfa root cultures expressing barley hemoglobin. Planta 219:95–102. 10.1007/s00425-003-1192-3 14740214 10.1007/s00425-003-1192-3

[CR34] Igamberdiev AU, Bykova NV, Shah JK, Hill RD (2010) Anoxic nitric oxide cycling in plants: participating reactions and possible mechanisms. Physiol Plant 138:393–404. 10.1111/j.1399-3054.2009.01314.x 19929898 10.1111/j.1399-3054.2009.01314.x

[CR35] Jasid S, Simontacchi M, Bartoli CG, Puntarulo S (2006) Chloroplasts as a nitric oxide cellular source. Effect of reactive nitrogen species on chloroplastic lipids and proteins. Plant Physiol 142:1246–1255. 10.1104/pp.106.086918 16980561 10.1104/pp.106.086918 PMC1630751

[CR36] Jeandroz S, Wipf D, Stuehr DJ, Lamattina L, Melkonian M, Tian Z, Zhu Y, Carpenter EJ, Wong GK-S, Wendehenne D (2016) Occurrence, structure, and evolution of nitric oxide synthase-like proteins in the plant kingdom. Sci Signal 9:417 re2. 10.1126/scisignal.aad4403 26933064 10.1126/scisignal.aad4403

[CR37] Kim-Shapiro DB, Gladwin MT (2014) Mechanisms of nitrite bioactivation. Nitric Oxide 38:58–68. 10.1016/j.niox.2013.11.002 24315961 10.1016/j.niox.2013.11.002 PMC3999231

[CR38] Kolbert Z, Barosso JB, Brouquisse R, Corpas FJ, Gupta KJ, Lindermayr C, Loake GJ, Palma JM, Petřvalský M, Wendehenne D, Hanock JT (2019) A forty year journey: generation and roles of no in plants. Nitric oxide 93:53–70. 10.1016/j.niox.2019.09.006 31541734 10.1016/j.niox.2019.09.006

[CR39] León J, Costa-Broseta Á (2020) Present knowledge and controversies, deficiencies, and misconceptions on nitric oxide synthesis, sensing, and signaling in plants. Plant Cell Environ 43:1–15. 10.1111/pce.13617 10.1111/pce.13617 31323702

[CR40] Liu B, Rennenberg H, Kreuzwieser J (2015) Hypoxia induces stem and leaf nitric oxide (NO) emission from poplar seedlings. Planta 241:579–589. 10.1007/s00425-014-2198-8 25398429 10.1007/s00425-014-2198-8

[CR41] Neill SJ, Desikan R, Hancock JT (2003) Nitric oxide signalling in plants. New Phytol 159:11–35. 10.1046/j.1469-8137.2003.00804.x 33873677 10.1046/j.1469-8137.2003.00804.x

[CR42] Newton AC, Flavell AJ, George TS, Leat P, Mullholland B, Ramsay L, Revoredo-Giha C, Russell J, Steffenson BJ, Swanston JS, Thomas WTB, Waugh R, White PJ, Bingham IJ (2011) Crops that feed the world 4. Barley: a resilient crop? Strengths and weaknesses in the context of food security. Food Sec 3:141–178. 10.1007/s12571-011-0126-3

[CR43] Nishimura H, Hayamizu T, Yanagisawa Y (1986) Reduction of NO_2_ to NO by rush and other plants. Environ Sci Technol 20:413–416. 10.1021/es00146a017 22300216 10.1021/es00146a017

[CR44] Perazzolli M, Romero-Puertas MC, Delledonne M (2006) Modulation of nitric oxide bioactivity by plant haemoglobins. J Exp Bot 57:479–488. 10.1093/jxb/erj051 16377734 10.1093/jxb/erj051

[CR45] Planchet E, Gupta KJ, Sonoda M, Kaiser WM (2005) Nitric oxide emission from tobacco leaves and cell suspensions: rate limiting factors and evidence for the involvement of mitochondrial electron transport. Plant J 41:732–743. 10.1111/j.1365-313X.2005.02335.x 15703060 10.1111/j.1365-313X.2005.02335.x

[CR46] Recalde L, Mansur NMG, Cabrera AV, Matayoshi CL, Gallego SM, Groppa MD, Benavides MP (2021) Unravelling ties in the nitrogen network: Polyamines and nitric oxide emerging as essential players in signalling roadway. Ann Appl Biol 178:192–208. 10.1111/aab.12642

[CR47] Rockel P, Rockel A, Wildt J, Segschneider HJ (1996) Nitric oxide (NO) emission by higher plants. In: Van Cleemput O, Hofman G, Vermoesen A (Eds.), Progress in nitrogen cycling studies. Developments in Plant and Soil Sciences, 68. Springer, Kluwer Academic Publishers, Dordrecht, 603–606. 10.1007/978-94-011-5450-5_98

[CR48] Saisho D, Takeda K (2011) Barley: Emergence as a new research material of crop science. Plant Cell Physiol 52:724–727. 10.1093/pcp/pcr049 21565909 10.1093/pcp/pcr049

[CR49] Schmidt K, Mayer B (2004) Consumption of nitric oxide by endothelial cells: Evidence for the involvement of a NAD(P)H-, flavin- and heme-dependent dioxygenase reaction. FEBS L 577:199–204. 10.1016/j.febslet.2004.10.010 10.1016/j.febslet.2004.10.010 15527785

[CR50] Stöhr C, Ullrich WR (1997) A succinate-oxidising nitrate reductase is located at the plasma membrane of plant roots. Planta 203:129–132. 10.1007/s00050173

[CR51] Stöhr C, Ullrich WR (2002) Generation and possible roles of NO in plant roots and their apoplastic space. J Exp Bot 53:2293–2303. 10.1093/jxb/erf110 12432022 10.1093/jxb/erf110

[CR52] Stöhr C, Strube F, Marx G, Ullrich WR, Rockel P (2001) A plasma membrane-bound enzyme of tobacco roots catalyses the formation of nitric oxide from nitrite. Planta 212:835–841. 10.1007/s004250000447 11346959 10.1007/s004250000447

[CR53] Stoimenova M, Igamberdiev AU, Gupta KJ, Hill RD (2007) Nitrite-driven anaerobic ATP synthesis in barley and rice root mitochondria. Planta 226:465–474. 10.1007/s00425-007-0496-0 17333252 10.1007/s00425-007-0496-0

[CR54] Tischner R, Planchet E, Kaiser WM (2004) Mitochondrial electron transport as a source for nitric oxide in the unicellular green alga *Chlorella sorokiniana*. FEBS L 576:151–155. 10.1016/j.febslet.2004.09.004 10.1016/j.febslet.2004.09.004 15474028

[CR55] Welle M, Niether W, Stöhr C (2024) The underestimated role of plant root nitric oxide emission under low-oxygen stress. Front Plant Sci 15:1290700. 10.3389/fpls.2024.1290700 38379951 10.3389/fpls.2024.1290700 PMC10876902

[CR56] Wildt J, Kley D, Rockel A, Rockel P, Segschneider HJ (1997) Emission of NO from several higher plant species. J Geophys Res Atmos 102:5919–5927. 10.1029/96JD02968

[CR57] Zhang J, Buegger F, Albert A, Ghirardo A, Winkler B, Schnitzler J-P, Hebelstrup KH, Durner J, Lindermayr C (2019) Phytoglobin overexpression promotes barley growth in the presence of enhanced level of atmospheric nitric oxide. J Exp Bot 70:4521–4537. 10.1093/jxb/erz249 31245808 10.1093/jxb/erz249 PMC6736386

[CR58] Zs K, Barosso JB, Brouquisse R, Corpas FJ, Gupta KJ, Lindermayr C, Loake GJ, Palma JM, Petřvalský M, Wendehenne D, Hanock JT (2019) A forty year journey: Generation and roles of NO in plants. Nitric Oxide 93:53–70. 10.1016/j.niox.2019.09.006 31541734 10.1016/j.niox.2019.09.006

