# Supplementary figures and images for: Inhibition of flavohemeproteins enhances the emission and level of nitric oxide in barley root tips
Source: Protoplasma. 2025 Apr 1;262(5):1195–206. doi: 10.1007/s00709-025-02058-w (PMC12394348; doi:10.1007/s00709-025-02058-w)

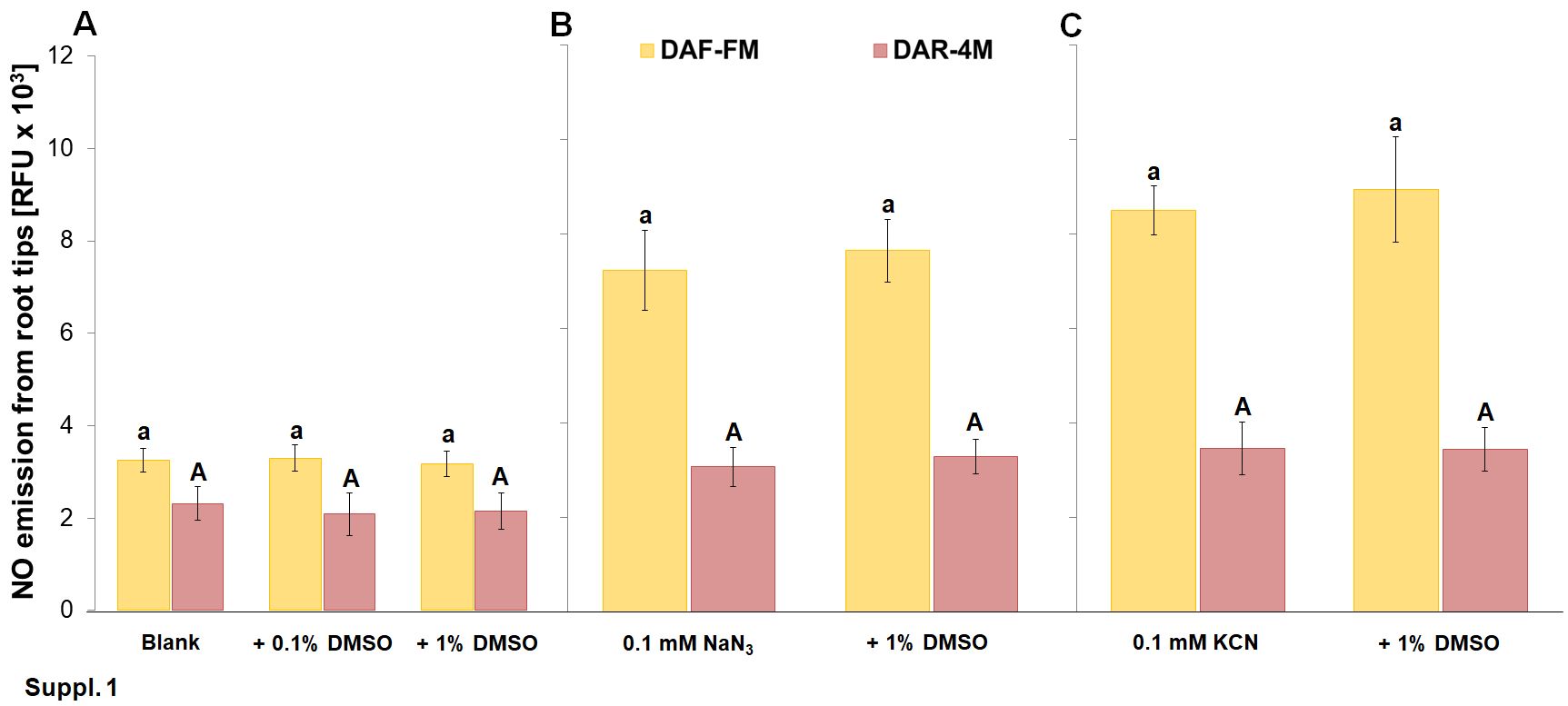

Supplement: Supplementary file 1 — Supplementary file1 (JPG 63 KB) [file 709_2025_2058_MOESM1_ESM.jpg]

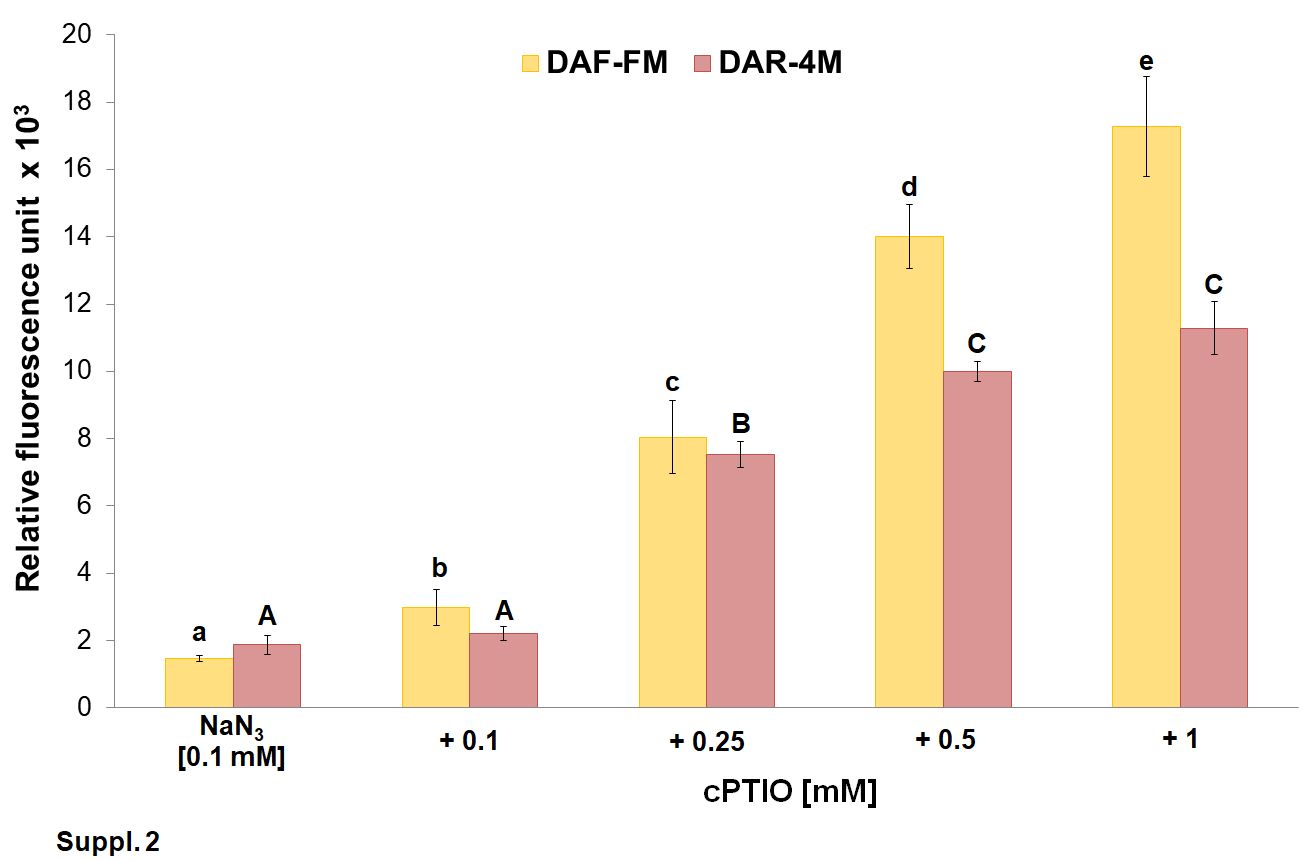

Supplement: Supplementary file 2 — Supplementary file2 (JPG 48 KB) [file 709_2025_2058_MOESM2_ESM.jpg]

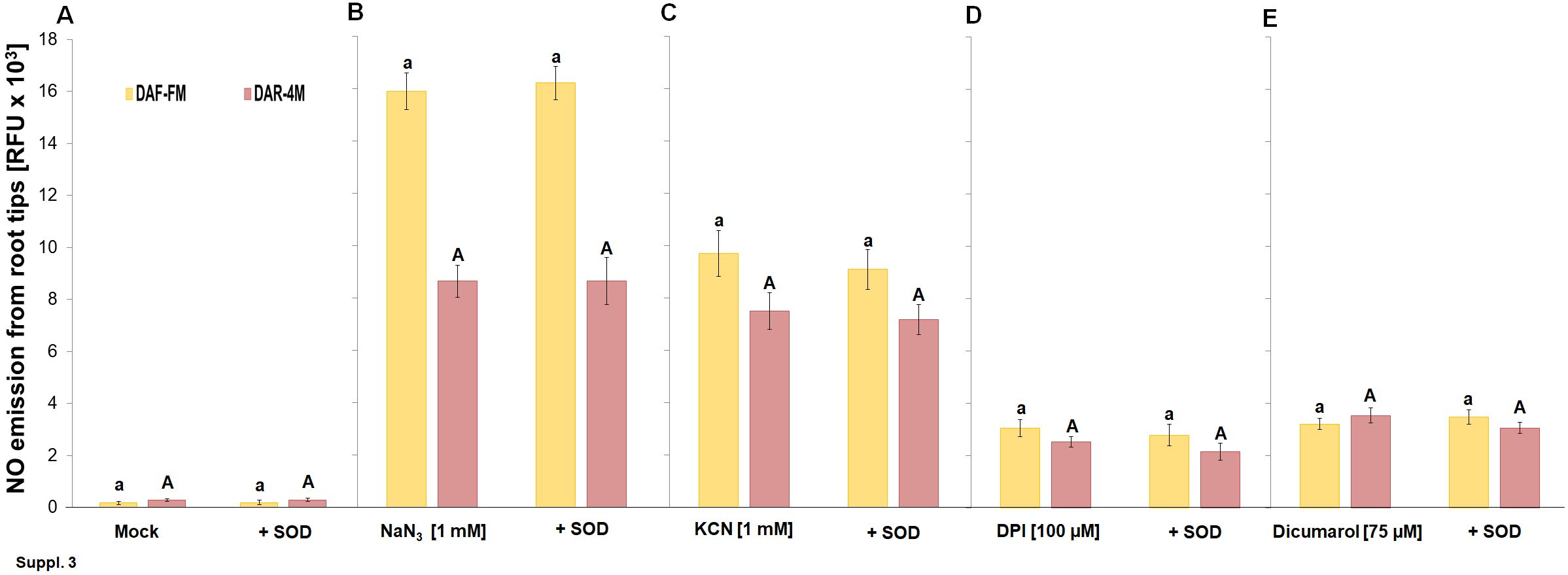

Supplement: Supplementary file 3 — Supplementary file3 (JPG 89 KB) [file 709_2025_2058_MOESM3_ESM.jpg]

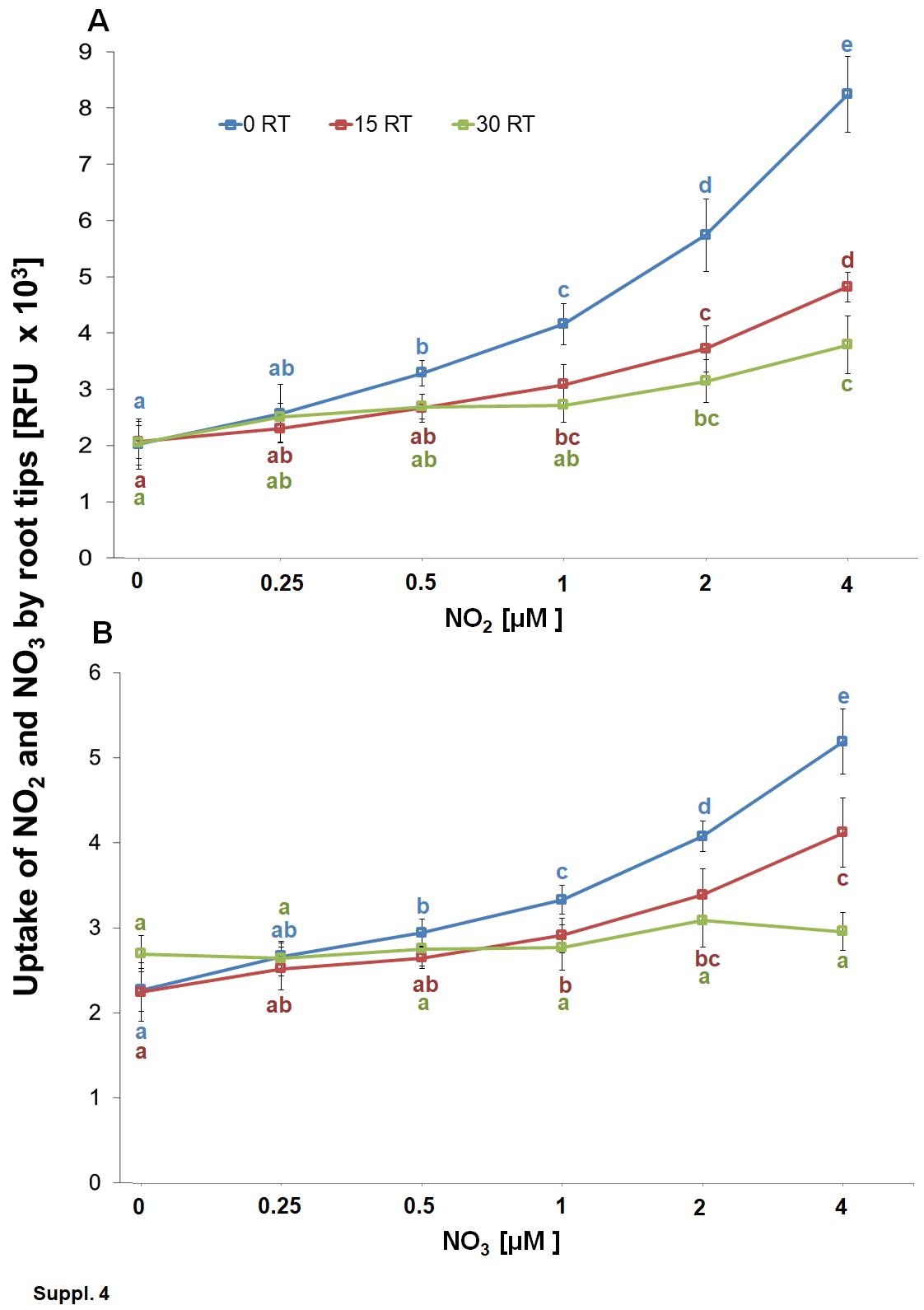

Supplement: Supplementary file 4 — Supplementary file4 (JPG 89 KB) [file 709_2025_2058_MOESM4_ESM.jpg]

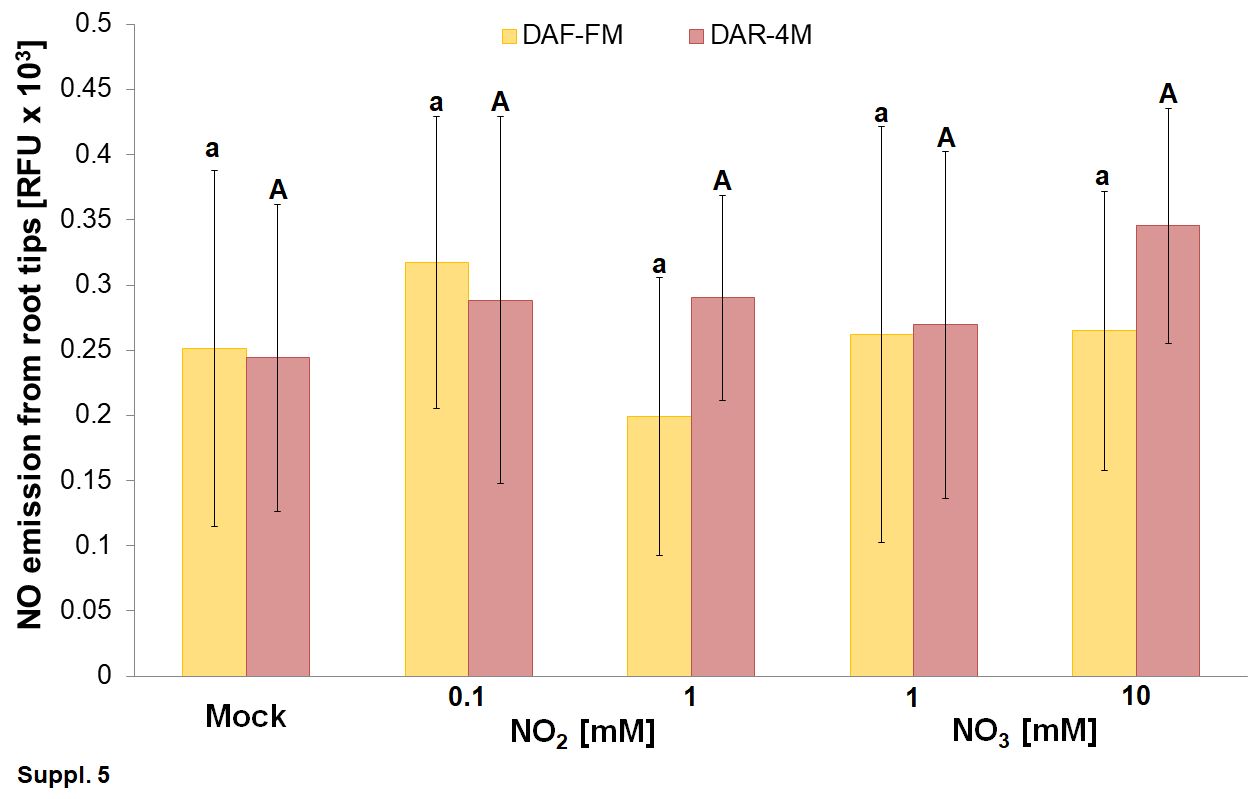

Supplement: Supplementary file 5 — Supplementary file5 (JPG 55 KB) [file 709_2025_2058_MOESM5_ESM.jpg]

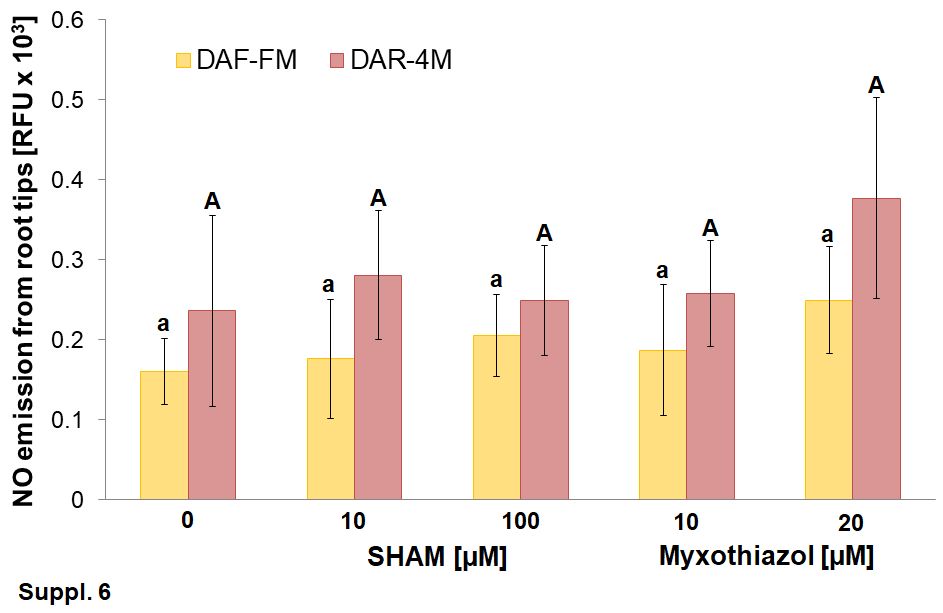

Supplement: Supplementary file 6 — Supplementary file6 (JPG 39 KB) [file 709_2025_2058_MOESM6_ESM.jpg]
